# Effect of Type of Dialysis on CMV-Specific CD8+ T Cells in Kidney Transplant Candidates

**DOI:** 10.3389/fimmu.2019.01680

**Published:** 2019-07-19

**Authors:** Jose Ramón Vidal-Castiñeira, Viviana Corte-Iglesias, Lucia Sobrino-Diaz, Sonia Pérez-Fernández, Santiago Melón, Carlos López-Larrea, Carmen Díaz-Corte

**Affiliations:** ^1^Translational Immunology Laboratory, Health Research Institute of the Principality of Asturias, Hospital Universitario Central de Asturias, Oviedo, Spain; ^2^Nephrology Service, Hospital Universitario Central de Asturias, Oviedo, Spain; ^3^Department of Statistics and Operational Research and Mathematics Didactics, Universidad de Oviedo, Oviedo, Spain; ^4^Microbiology Service, Hospital Universitario Central de Asturias, Oviedo, Spain; ^5^Immunology Service, Hospital Universitario Central de Asturias, Oviedo, Spain

**Keywords:** CMV, hemodialysis, peritoneal dialysis, CMV-specific CD8+ T cells, dextramers

## Abstract

**Background:** Dialysis is the first procedure to partially replace renal function in end-stage renal diseases, despite several adverse side effects, such as infections. The primary aim of this study was to evaluate the levels of immune CMV-specific CD8+ T cells in a representative cohort of pre-transplant patients receiving hemodialysis (HD) or peritoneal dialysis (PD). The secondary aim was to monitor the CMV-specific CD8+ T cells in kidney transplant recipients undergoing different types of dialysis during the first year following their transplant.

**Methods:** Sixty-nine patients were enrolled and examined with respect to the type of dialysis they received. HLA class I dextramers for CMV were used to determine the quantity of CMV-specific CD8+ T cells. The CMV DNA viral load was also determined. Forty-two of the patients enrolled in the study underwent solid organ transplantation and were analyzed during their first year post-transplantation.

**Results:** Patients receiving HD had fewer CMV-specific CD8+ T cells than those in PD (*p* < 0.05). We also observed that patients in PD had more CMV-specific CD8+ T cells during the follow-up period than those in HD (*p* < 0.05), independently of the CMV DNA. Finally, PD patients had a higher frequency of CD8+ Effector-Memory RA T cells (TEMRA) and a lower frequency of central memory T cells (TCM) than did HD patients.

**Conclusions:** These results indicate the better status of CMV-specific T cell immunity in PD patients. The use of CMV T cell dextramers would be advantageous for monitoring the CD8+ T-specific response, enabling the use of prophylactic treatment to be optimized.

## Introduction

Patients in end-stage renal diseases experience disturbances of the immune system and are highly susceptible to infections arising from dialysis. The frequency of mortality of patients in dialysis is higher than in the general population, especially in patients in hemodialysis (HD) compared with those in peritoneal dialysis (PD) (1, 2). The risk of infections in these patients increases following kidney transplantation, especially during the first 12 months, because of the initial immunosuppression, which makes the recipient susceptible to serious infections such as human cytomegalovirus (CMV) (3). Primary infection or reactivation with CMV may cause a viremia and can lead to severe CMV disease with organ involvement (4). The clinical manifestations of human CMV infection include CMV syndrome (viremia and neutropenia), graft infection, predisposition to opportunistic infections, post-transplant lymphoproliferative disorders (generally with Epstein–Barr virus [EBV]), and chronic effects, including accelerated vasculopathy (5).

Two main strategies are used to prevent CMV infection after kidney organ transplantation (6). The first is prophylaxis of viral infections using antiviral drugs; the second strategy is preemptive therapy for organ recipients who develop evidence of CMV infection during routine screening (7). These strategies have brought about significant reductions of CMV infection and CMV-related mortality (8) but, on the other hand, they have led to a higher risk of developing anti-CMV drug resistance, a higher cost of antiviral medication, and a greater risk of side effects, with many patients being over-treated (9).

Customarily, CMV-DNA copy number has been used to identify viral reactivation (10), but the cellular immunity mediated by T lymphocytes is more relevant for controlling CMV infection (11). Immunity to CMV depends on the provision of adequate help from CMV-specific CD4+ T cells that enable production of neutralizing antibodies by CMV-specific B-cells/plasma blasts, and effective cytotoxic CD8+ T cell (CTL) responses (12). Many studies have reported the relationship between post-transplant functional impairment of CD8+ T cells immunity and failure to suppress CMV replication after kidney transplantation (8, 13), but little is known about the influence of the type of dialysis on CMV-specific CD8+ T cell status. Most of them have focused on evaluating functional immune responses (14, 15).

The aim of this study was to analyze the impact of the type of dialysis on the frequency of CMV-specific CD8+ T cells in pre- and post-transplant patients. Our overall purpose was to redefine therapeutic strategies for selected groups of immunosuppressed patients.

## Methods

### Study Population

A cohort of 69 unrelated Caucasian patients on the waiting list for a kidney transplant in the Nephrology Service of the Hospital Universitario Central de Asturias (HUCA), Oviedo, Spain, were included in the study between February 2016 and February 2017 (Table 1). They were recruited when they were called for organ transplantation. Forty-two of these patients (61.9% males) received a kidney transplant and were followed up for 1 year. Comprehensive clinical and analytical data of these patients were collected in order to analyze the evolution of the CMV-CD8+ specific T cells.

**Table 1:** Baseline clinical characteristics of the patient groups.

**Characteristics**	**Patients analyzed (*n* = 69)**	**Patients in HD (*n* = 41)**	**Patients in PD (*n* = 26)**	**Pre-dialysis (*n* = 2)**
Age, median (Range), years	63 (29–80)	64 (29–75)	60.5 (31–74)	76.5 (73–80)
**Gender distribution [*****n*** **(%)]**				
Male	45 (65.2)	32 (78)	12 (46.2)	1 (50)
Female	24 (34.8)	9 (22)	14 (53.8)	1 (50)
Time on dialysis, median (Range), months	43.9 (3.7–247)	43.6 (3.7–247)	47 (8–109.2)	–
**Underlying kidney disease [*****n*** **(%)]**				
Polycystic kidney disease	13 (18.9)	8 (19.5)	5 (19.2)	0 (0)
Primary glomerulopathies	22 (31.9)	15 (36.6)	7 (26.9)	0 (0)
Nephrosclerosis/atherosclerosis/hypertension	9 (13)	5 (12.2)	3 (11.6)	1 (50)
Diabetes	4 (5.8)	3 (7.3)	1 (3.9)	0 (0)
Other	12 (17.4)	6 (14.6)	5 (19.2)	1 (50)
Unknown	9 (13)	4 (9.8)	5 (19.2)	0 (0)
**CMV serological status [n (%)]**				
CMV+	59 (85.5)	35 (85.4)	22 (84.6)	2 (100)
CMV–	10 (14.5)	6 (14.6)	4 (15.4)	0 (0)

*At least one HLA class I of the dextramers was analyzed in all patients. HD, Hemodialysis; PD, Peritoneal dialysis*.

Blood samples were extracted in a blood collection tube with EDTA at different times (pre-transplant, and 15, 30, 45, 60, 90, 120, 150, 180, 210, 240, 270, 300, and 360 days post-transplant).

This study was carried out in accordance with the recommendations of the Regional Ethics Committee of Clinical Research of the Principado de Asturias. All the subjects gave their written informed consent in accordance with the Declaration of Helsinki. The protocol was approved by the Ethics Committee of our hospital.

### CMV Viral Load Determination

CMV viral serostatus of the patients was determined as part of current routine clinical practice in the Microbiology Service (HUCA) using the Liaison CMV IgG II assays (DiaSorin, Saluggia, Italy). In-house real-time PCR was used to quantify CMV DNA in accordance with general regulatory guidelines concerning CMV viral load monitoring. A blood sample was extracted in a blood collection tube with EDTA, and the viral DNA was isolated from peripheral blood mononuclear cells (PBMCs). The viral β-glycoprotein and the human APOB gene as a control were amplified and hybridized with fluorescent-labeled probes in an RT-PCR based on TaqMan Genotyping Assays (Applied Biosystems, Foster City, CA, USA). The viral load in copies of CMV DNA per mL was determined, from which the International Units (IU) per mL were then calculated. The CMV DNA data of the patients were collected at the same post-transplant times as for the CMV-specific CD8+ T cell determination.

### Flow Cytometry Analysis

The Dextramer CMV kit (Immudex, Copenhagen, Denmark) was used according to the manufacturer's instructions to quantify the CMV-specific CD8+ T cells. The CMV-MHC class I dextramers used, which covered most HLA specificities, were A:0101, A:0201, A:0301, A:2402, B:0702, B:0801, and B:3501. Samples were acquired using a Gallios flow cytometer (Beckman Coulter, Pasadena, CA, USA) and analyzed using Kaluza Analysis software (Beckman Coulter, Pasadena, CA, USA). A whole-blood control for lymphocyte subset enumeration was used in all sessions (BD^TM^ Multi-Check control, BD Biosciences, San Jose, CA, USA). Absolute CD8+ T cells number and percentage were calculated using Trucount Tubes (BD Biosciences, San Jose, CA, USA).

For CD8+ T cell phenotyping, whole-EDTA anticoagulate was obtained by venipuncture. Peripheral blood mononuclear cells were obtained by Ficoll–Paque density-gradient centrifugation and directly analyzed. Naïve (CCR7+CD45RA+), central memory (CM) (CCR7+CD45RA–), effector memory (EM) (CCR7–CD45RA−), and effector memory RA (EMRA) (CCR7–CD45RA+) CD8+ T cells were assessed by flow cytometry using the following antibodies: CD3 (PerCP; BD Biosciences, San Jose, CA, USA), CD8 (FITC; BD Biosciences, San Jose, CA, USA), CD45RA (ECD; Beckman Coulter, Pasadena, CA, USA), and CCR7 (APC; BioLegend, San Diego, CA, USA).

### ELISpot Assay for IFN-γ Detection of CMV-Specific T Cells

CMV-specific T cell activity was determined by measuring IFN-γ upon stimulation of PBMCs in 43 patients. The PBMCs were isolated from 5 mL of citrate blood using a standard Ficoll–Paque density gradient and resuspended in RPMI 1640 medium containing 2 ×10^−3^M l-glutamine and Hepes, supplemented with 10% FCS (ICN Flow, Costa Mesa, CA, USA) and antibiotics. For the ELISpot assay, 3 ×10^5^ PBMCs per well were placed in triplicate wells of a 96-well filter plate (Millipore, Billerica, MA, USA) that was coated with anti-IFN-γ Ab (BD Biosciences, San Jose, CA, USA). These cells were stimulated with CMV-pp65 (PM-PP65-1; JPT, Berlin, Germany) and with 1 μg/mL anti-PMA (Sigma-Aldrich, St. Louis, MO, USA) as a positive control, and incubated for 24 h at 37°C. Negative controls were also included using PBMCs plus medium and DMSO. Plates were incubated for 2h at room temperature with 100 μL (1 μL/mL) biotinylated detection IFN-γ antibody to detect IFN-γ captured by the plate-bound Ab. Subsequently, plates were incubated with streptavidin (1 μg/mL) for 2 h at room temperature. As a final step, spots were developed by adding 200 μL of 3-amino-9-ethylcarbazole (BD Biosciences, San Jose, CA, USA) in acetate buffer supplemented with H_2_O_2_ 30% for 3–5 min. Resulting spots were counted using a computer-assisted ELISpot reader (AID Autoimmun Diagnostika GmbH, Strassberg, Germany). Positive ELISpot signals were defined as those containing at least 25 spot-forming units per well.

### Statistical Analysis

A descriptive statistical analysis of the variables was done separately for pre-transplant and post-transplant patients who received HD or PD. The continuous numerical variables were not normally distributed, so they were summarized as their median and range. Absolute and relative frequencies are displayed for qualitative variables. In order to test the difference between dialysis groups, the non-parametric Wilcoxon rank-sum test was used for continuous measures, while Fisher's exact test was considered for qualitative variables.

Multiple linear and logistic regression models were fitted as a multivariate study of the simultaneous statistical associations among several of the variables studied. Model terms were chosen in a backwards-stepwise manner, considering the Akaike information criterion (AIC), and the parameter estimates with 95% confidence intervals and associated probabilities were reported for the final model. The distribution of those measurements showing statistically significant differences between groups was displayed using boxplots. Statistical significance was concluded for values of *p* < 0.05 in all analyses. Data were analyzed within the R statistical environment (version 3.6.0).

## Results

### Analysis of Patients on the Waiting List

**Figure 1:**
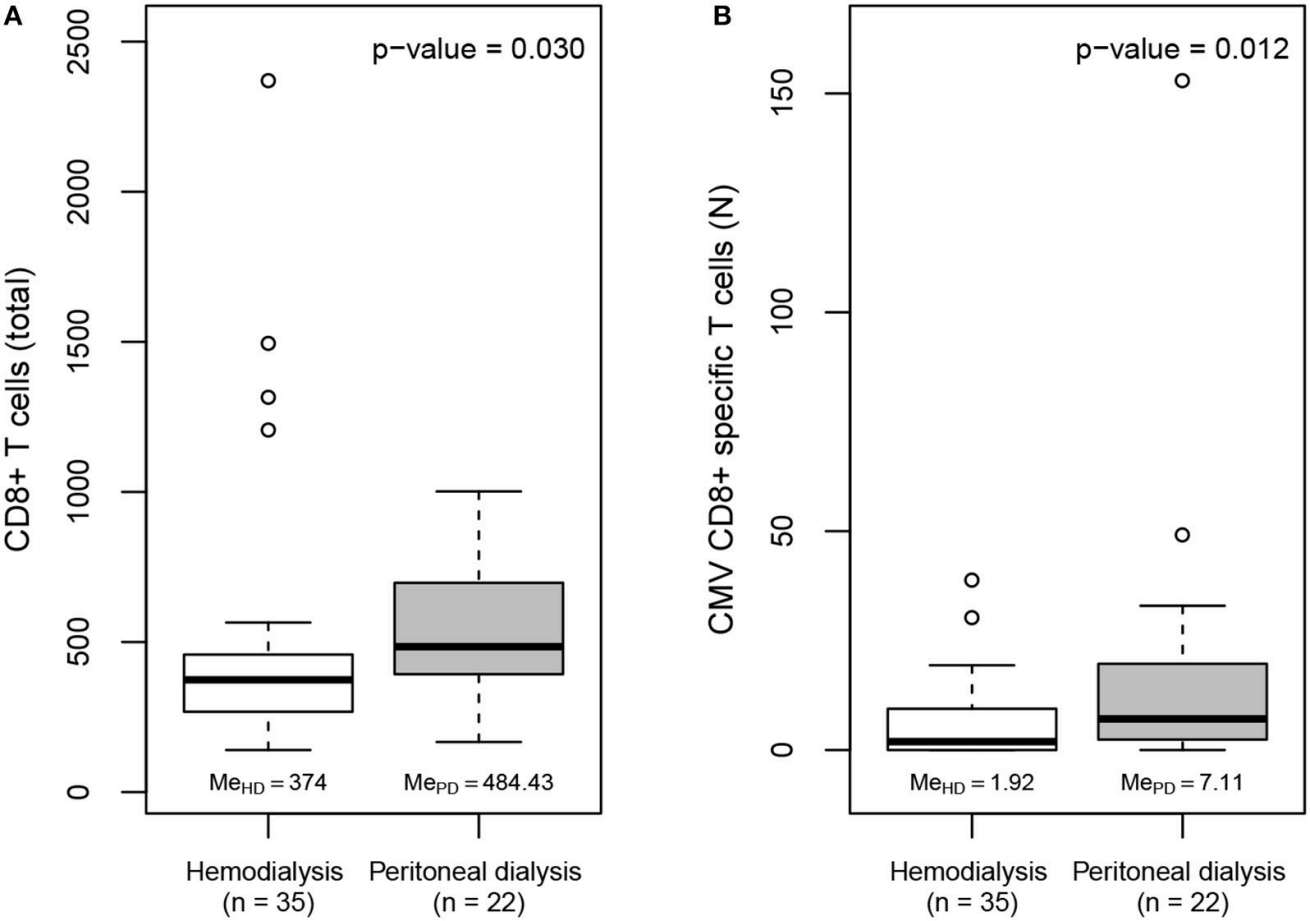
**(A)** Boxplots of the total frequency of CD8+ T cells in CMV seropositive patients on the kidney transplant waiting list by dialysis group. **(B)** Boxplots of the total frequency of CMV-specific CD8+ T cells in CMV seropositive patients on the kidney transplant waiting list by dialysis group.

Patients were classified according to the type of dialysis they were receiving (*n* = 69; Table 1): 59.4% of them were in HD and 37.7% were in PD. The two other patients (2.9%) were included on the waiting list for kidney transplant in pre-dialysis because of the clinical recommendation of the nephrologists. The number of CMV-specific CD8+ T cells in the pre-transplant state was studied with respect to the patients' dialysis type. We found no significant differences between the dialysis groups in the total number of CD8+ T cells. However, there were statistically significant differences between them with respect to the proportions of patients who were serologically CMV+ (*p* = 0.03; Figure 1A). Among these CMV+ patients, we observed that those in HD had fewer CMV-specific CD8+ T cells than those receiving PD (median: HD vs. PD, 1.92 vs. 7.11, *p* = 0.012; Figure 1B).

Multivariate linear regression models were derived with the log-transformed frequency of CMV-specific CD8+ T cells as the response variable, and the type of dialysis and variables related to anti-CMV immunity (age, time on dialysis, serological CMV status and absolute frequency of CD8+ T cells) as regressors in the initial model. The final stepwise regression model shows that the type of dialysis (*p* < 0.01) and serological CMV status (*p* < 0.005) were significantly related to the frequency of anti-CMV CD8+ T cells (Table 2).

**Table 2:** Multiple linear regression model to explain variation in CMV-specific CD8+ T cell frequency.

**Covariates**	**Coefficient estimate (95% CI)**	***p***
Dialysis (Peritoneal dialysis)	0.86 (0.26, 1.47)	<0.01
Serological CMV status (CMV–)	−1.4 (−2.24, −0.58)	<0.005

*Covariates initially considered in the model were: type of dialysis, age, time on dialysis, serological CMV status, and absolute CD8+ T cell frequency. Regression coefficients with 95% confidence intervals and associated probabilities are shown for the significant terms in the final model. The overall model significance was p < 0.001 (F-test)*.

In relation to the CD8+ T cells subsets, we found that patients in PD tended to have a higher porcentage of CD8+ TEMRA cells (median [range]: PD vs. HD, 48.3 [13–67] vs. 29.9 [17–72]) and a lower frequency of TCM cells than patients in HD (median [range]: HD vs. PD, 16.3 [6.2–31] vs. 7.8 [3.1–27]), but the magnitude of the difference was not sufficiently great to be statistically significant in the small sample of patients analyzed. Finally, in order to determine the immune responsiveness of these specific T cells we analyzed the levels of pre-transplant IFN-γ using a CMV ELISpot assay. Patients in HD exhibited preformed CMV-specific T cells directed to CMV-pp65 in 73.9% of the cases analyzed (17/23 of the patients, median frequency of spots/3 ×10^5^ PBMCs [range], 77 [3–233]) compared with those in PD, 85% of whom (17/20, median frequency of spots/3 ×10^5^ PBMCs [range], 94 [7–334]) showed preformed CMV-specific T cells directed to CMV-pp65. These differences were not significant.

In conclusion, we observed that the frequency of CMV-specific CD8+ T cells was higher in patients in PD than in those on HD. These patients also had a better cellular immune status against CMV than those in HD.

### Analysis of Transplant Patients

We followed up those patients who had undergone solid organ transplantation (*n* = 42) for 1 year. Of the 42 patients, 19 experienced CMV reactivation (>100 IU/mL) within 360 days post-transplantation (Table 3), and five appeared to have no CMV-specific CD8+ T cells during the follow-up period after transplantation. Three of these five recipients were seronegative for CMV just before the kidney transplant but received a CMV-seropositive transplant. The other 37 patients were positive for CMV-specific CD8+ T cells on one or more occasion.

**Table 3:** Characteristics of transplanted patients by dialysis type.

**Characteristics**	**Transplanted patients (*n* = 42)**	**Patients in hemodialysis (*n* = 21)**	**Patients in peritoneal dialysis (*n* = 20)**	**Patients in pre-dialysis (*n* = 1)**
Age, median (Range), years	64 (29–80)	67 (29–76)	60.5 (41–74)	80
**Gender distribution [*****n*** **(%)]**				
Male	26 (61.9)	16 (76.2)	9 (45)	1 (100)
Female	16 (38.1)	5 (23.8)	11 (55)	0 (0)
**Type of donor [*****n*** **(%)]**				
Deceased	40 (95.2)	20 (95.2)	19 (95)	1 (100)
Living	2 (4.8)	1 (4.8)	1 (5)	0 (0)
**CMV Serological status [*****n*** **(%)]**				
CMV+	38 (90.5)	19 (90.5)	18 (90)	1 (100)
CMV–	4 (9.5)	2 (9.5)	2 (10)	0 (0)
**Receptor/donor CMV status [*****n*** **(%)]**				
R–/D–	1 (2.4)	1 (4.7)	0 (0)	0 (0)
R–/D+	3 (7.1)	1 (4.7)	2 (10)	0 (0)
R+/D–	6 (14.3)	2 (9.5)	4 (20)	0 (0)
R+/D+	32 (76.2)	17 (81.1)	14 (70)	1 (100)
**Induction therapy [*****n*** **(%)]**				
Thymoglobulin	5 (11.9)	3 (14.3)	2 (10)	0 (0)
Basiliximab	28 (66.7)	16 (76.2)	11 (55)	1 (100)
Triple conventional	9 (21.4)	2 (9.5)	7 (35)	0 (0)
**CMV treatment [*****n*** **(%)]**				
No treatment	24 (57.2)	11 (52.4)	13 (65)	0 (0)
Prophylaxis	1 (2.4)	1 (4.8)	0 (0)	0 (0)
Treatment	9 (21.4)	6 (28.6)	3 (15)	0 (0)
Prophylaxis+treatment	8 (19)	3 (14.3)	4 (20)	1 (100)
**CMV reactivation [*****n*** **(%)]**				
Yes	19 (45.2)	9 (42.9)	9 (455)	1 (100)
No	23 (54.8)	12 (57.1)	11 (55)	0 (0)
CMV-specific CD8+ T cells at time of transplantation, median (Range)	3.38 (0–153)	0 (0–38.75)	6.58 (0–153)	0
**Mean CMV-specific CD8+** T cells^*^, median (range)				
Total	12.03 (0–239.13)	6.73 (0–64.75)	13.97 (0–239.13)	14.81
CMV reactivation	14.81 (0–134.11)	6.73 (0–40.61)	22.15 (0–134.11)	14.81
CMV non-reactivation	11.34 (0–239.13)	7.64 (0–64.75)	12.9 (0.25–239.13)	
**CMV DNA copies/10**^**5**^ **leukocytes, median (range)**				
Maximum	234 (67–18653)	893 (67–18653)	154 (94–4805)	234
Mean^¥^	163 (67–6283)	299 (67–6283)	142 (90.5–1686)	156
**CMV DNA IU/mL, median (range)**				
Maximum	2233.8 (709.6–197724.2)	5911.8 (709.6–197724.2)	1570.6 (765.8–25945.4)	2344.6
Mean^+^	2028.9 (709.6–56868)	3369.1 (709.6–56868)	1465.8 (763.1–9841.5)	1705.7

* *Total CMV-specific CD8+ T cell frequency during follow-up period divided by 15 (number of time-points considered in the study)*.

¥ *Total frequency of CMV DNA copies/10^5^ leukocytes during follow-up period divided by 15 (number of time-points considered in the study). Patients with CMV reactivation analyzed*.

+ *Total frequency of CMV IU/mL during follow-up period divided by 15 (number of time-points considered in the study). Patients with CMV reactivation analyzed*.

**Figure 2:**
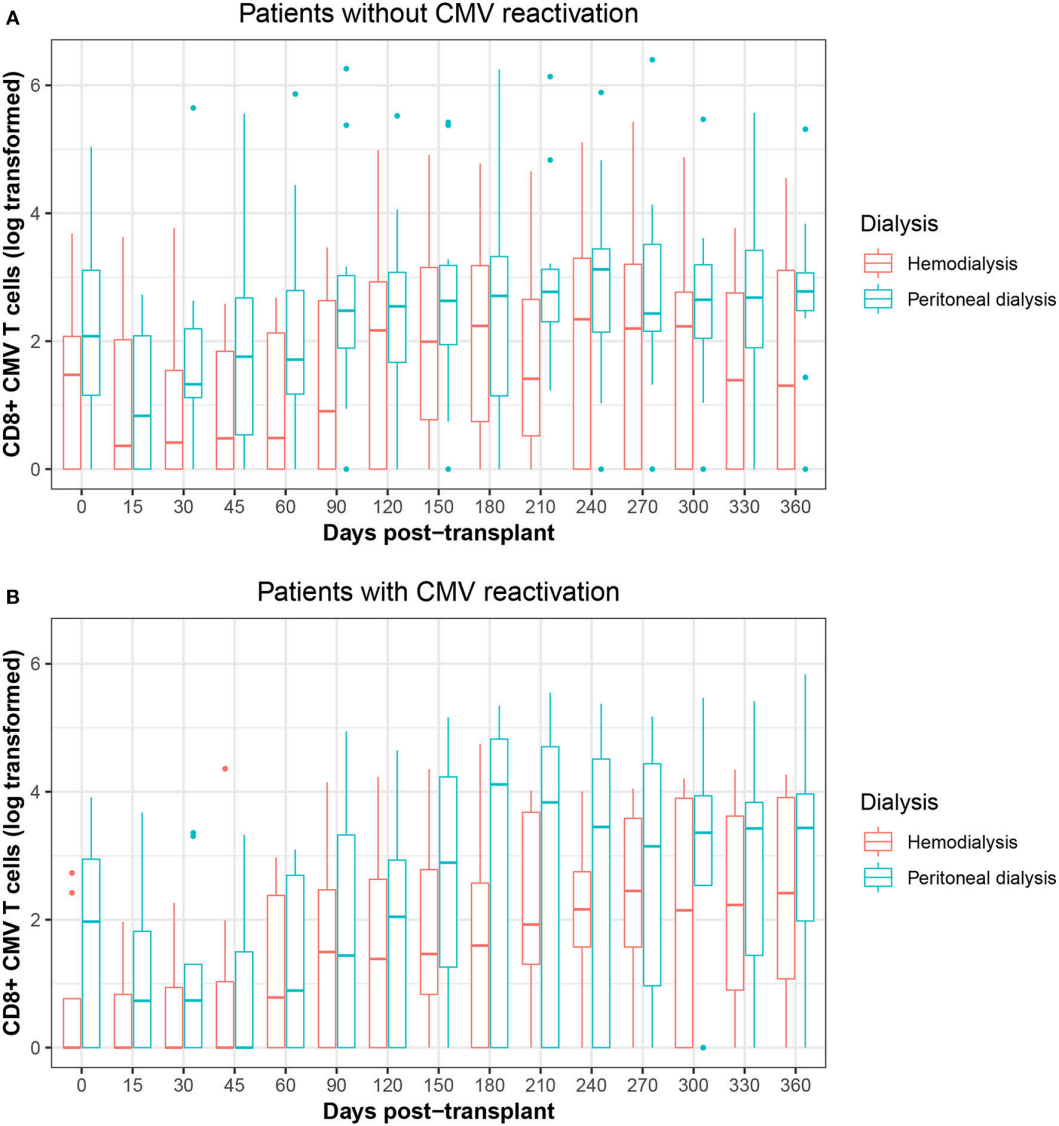
**(A)** Boxplots of the frequency of CMV-specific CD8+ T cells during the follow-up period classified by dialysis type in CMV non-reactivation patients. **(B)** Boxplots of the frequency of CMV-specific CD8+ T cells during the follow-up period classified by dialysis type in CMV reactivation patients.

The frequency of CMV-specific CD8+ T cells at the time of transplantation was higher in patients in PD compared with those in HD (PD vs. HD, median, 6.58 vs. 0, *p* = 0.026)—a similar finding to that noted in pre-transplanted patients. Furthermore, during the follow-up period, patients who were receiving PD had significantly more CMV-specific CD8+ T cells than did those in HD (PD vs. HD, median, 13.97 vs. 6.73, *p* = 0.015). The differences were greatest after 90 days, but frequencies had equalized by 360 days. Considering the patients with respect to the CMV reactivation, the CMV-specific CD8+ T cells frequency at the times studied showed that values for PD patients were more variable, but generally higher, than those patients in HD in both patient groups (Table 3, Figures 2A,B).

On the other hand, we found no statistically significant correlation between the frequency of CMV-specific CD8+ T cells and the amount of CMV DNA, although 36.8% (7 of 19) of the patients who experienced CMV reactivation and needed antiviral treatment were from the group who had received HD, compared with 26.2% (5 of 19) of the patients who had received PD. There were fewer than 5 CMV-specific CD8+ T cells/μl in this group at the beginning of the CMV reactivation. In contrast, 5 of 19 patients who underwent CMV reactivation did not receive antiviral prophylaxis or treatment. These patients (2 HD and 3 PD patients) had a high frequency of >20 CMV-specific CD8+ T cells/μl during follow-up.

Finally, other clinical parameters related to CMV reactivation, such as the CMV serostatus of the donors and recipients, the immunosuppressor levels and receipt of induction therapy were analyzed, but did not reveal any differences in the frequency of CMV-specific CD8+ T cells. A multivariate analysis including the type of dialysis, age, time on dialysis, serological CMV status, and total frequency of CD8+ T cells was performed, but none of the variables proved to be significantly associated with the increase in frequency of anti-CMV CD8+ T cells, possibly due to the low number of patients analyzed.

In summary, the frequency of CMV-specific CD8+ T cells increased in both groups of patients by 90 days, possibly due to the reduction in immunosuppression levels and the end of CMV prophylaxis. We also observed that patients in PD had better cellular immune status than those in HD.

## Discussion

CMV has a negative consequence for patients and allograft outcomes after kidney transplantation. CMV-specific CD8+ T cells are thought to be crucial to the control of CMV replication in the early phase after kidney transplantation (16). The frequency of CMV-specific CD8+ T cells has so far been little studied with regard to the risk of post-prophylaxis CMV infection, irrespective of the pre-transplant serological status. One study reported a strong positive correlation between the levels of CMV-specific CD4+ T cells and the frequency of infectious episodes in lung- but not kidney-transplant recipients, depending on the immunosuppression regimen employed (17). Moreover, Gordon et al. recently reported a correlation between the persistence of CMV and antiviral T cell immunity, whereby there was a different distribution of CMV-specific CD8+ T cells in several tissues (18).

The aim of this study was to monitor the frequency of CMV CD8+ specific T cells by using the dextramer technology in pre- and post-kidney transplant patients. We found more CMV-specific CD8+ T cells in patients in PD than in those receiving HD, and noted that this status was maintained throughout the 1-year follow-up after kidney transplantation, irrespective of patients' CMV serological status. Thus, protective anti-CMV cellular immunity may exist in these patients, unlike those receiving HD, indicating that patients in PD treatment were better able to resolve CMV reactivation. During the post-transplant follow-up, the presence of CMV-specific CD8+ T cells was not sufficient to prevent viral reactivation (CMV viremia) in most patients. Associated risk factors and a greater susceptibility to immunosuppressors led to the decline in the frequency of CMV-specific CD8+ T cells. This is insufficient during the initial months to provide further protection from CMV replication, and valganciclovir prophylaxis is necessary, at least for the first 3 months, in patients with risk factors such as D+/R– transplants. On the other hand, some patients with a high frequency of CMV-specific CD8+ T cells, most of whom are patients in PD, are able to control the viremia themselves without the need for antiviral treatment. Nevertheless, this variation between patients suggests that CMV-specific T cell monitoring could provide a means of risk stratification in addition to CMV serostatus.

The type of dialysis is known to have a range of effects on the immune response (19). Mild monocytosis and lymphopenia are more prevalent in patients receiving HD compared with those in PD, and these could be responsible for the higher rate of acute rejection episodes in the early stages following renal transplantation in patients in HD (20). Moreover, patients in HD are more prone to infections and to an inadequate response to vaccinations (21), and severe lymphopenia has been shown to be a predictor of mortality in these patients (19). Recently, a CD19+ B-cell lymphopenia was observed in HD patients, leading the authors to conclude that it could be a biomarker of cardiovascular complication-related mortality (22). In contrast, patients in PD were less likely to suffer chronic inflammations (23).

CMV infection generally leads to substantial and long-lasting changes in circulating T cells. In particular, those of the highly differentiated TEMRA (Effector Memory RA) cells subset are expanded in the CD8+ T cell population (24). Recently, Ferreira et al. have shown that organ transplant recipients who were unable to control CMV viremia had higher frequencies of TEMRA cells with low activation and differentiation markers compared with those with increased activation markers in TCM and TEMRA cells subsets and who were able to control their viremia (25). In this preliminary study, we observed differences in the CD8+ T cells subsets between patients in PD and HD, but the magnitude was not large enough to be statistically significant in the small sample of patients analyzed. Nevertheless, the description of the status of these CD8+ T cells subsets including other markers could help us understand how dialysis affects the maintenance of an adequate pool of the CMV-specific CD8+ T lymphocytes that are required to ensure immunity against CMV infection in the post-transplant context. In the near future, we will study more patients in order to verify our current results. As part of this work, we will analyze activation and differentiation markers in these CD8+ T cell subpopulations.

The determination of cellular immunity to assess risk before kidney transplantation by analyzing patients on the waiting list who are receiving dialysis, and the analysis of immune responsiveness using ELISpot, are powerful methods for evaluating CMV antiviral prophylaxis therapy. Further studies are needed to identify the variables associated with the risk of CMV reactivation, to characterize these CMV-specific T cells phenotypically, and to establish a cut-off for pre-transplant CMV-specific CD8+ T cells in order to be able to categorize patients.

## Data Availability

The raw data supporting the conclusions of this manuscript will be made available on request to the corresponding author.

## Ethics Statement

This study was carried out in accordance with the recommendations of the Regional Ethics Committee of Clinical Research of Principado de Asturias, with written informed consent from all subjects. The study was carried out in accordance with the Declaration of Helsinki. The protocol was approved by the Ethics committee of our hospital.

## Author Contributions

JV-C, CD-C, and CL-L: concept and design. JV-C, VC-I, LS-D, and SM: experiments and procedures. JV-C, VC-I, CD-C, and CL-L: writing of article. SP-F: statistical analysis. LS-D, SM, and CD-C: patients selection.

### Conflict of Interest Statement

The authors declare that the research was conducted in the absence of any commercial or financial relationships that could be construed as a potential conflict of interest.
